# Horizontal transfer of accessory chromosomes in fungi – a regulated process for exchange of genetic material?

**DOI:** 10.1038/s41437-025-00746-0

**Published:** 2025-02-10

**Authors:** Michael Habig, Satish Kumar Patneedi, Remco Stam, Henrik Hjarvard De Fine Licht

**Affiliations:** 1 Fungal Evolutionary Genetics, Christian-Albrechts University of Kiel, Kiel, Germany; 2 Phytopathology and Crop Protection, Christian-Albrechts University of Kiel, Kiel, Germany; 3 Section for Organismal Biology, Department of Plant and Environmental Sciences, University of Copenhagen, Copenhagen, Denmark

**Keywords:** Evolution, Epigenetics

## Abstract

Horizontal transfer of entire chromosomes has been reported in several fungal pathogens, often significantly impacting the fitness of the recipient fungus. All documented instances of horizontal chromosome transfers (HCTs) showed a marked propensity for accessory chromosomes, consistently involving the transfer of an accessory chromosome while other chromosomes were seldom, if ever, co-transferred. The mechanisms underlying HCTs, as well as the factors regulating the specificity of HCTs for accessory chromosomes, remain unclear. In this perspective, we provide an overview of the observed propensity in reported cases of horizontal chromosome transfers. We hypothesize the existence of a signal that distinguishes mobile, i.e., horizontally transferred, accessory chromosomes from the rest of the donor genome. Recent findings in *Metarhizium robertsii* and *Magnaporthe oryzae*, suggest that a mobile accessory chromosome may contain putative histones and/or histone modifiers, which could generate such a signal. Based on this, we propose that mobile accessory chromosomes may encode the machinery required for their own horizontal transmission, implying that HCT could be a regulated process. Finally, we present evidence of substantial differences in codon usage bias between core and accessory chromosomes in 14 out of 19 analysed fungal species and strains. Such differences in codon usage bias could indicate past horizontal transfers of these accessory chromosomes. Interestingly, HCT was previously unknown for many of these species, suggesting that the horizontal transfer of accessory chromosomes may be more widespread than previously thought, and therefore an important factor in fungal genome evolution.

Horizontal (lateral) gene transfer (HGT) is characterized as “the movement of genetic information across normal mating barriers between more or less distantly related organisms” (Keeling and Palmer, 2008). In the past decades, the discovery of widespread HGT has completely changed our view of prokaryote evolution, where in some species up to 80% of the genes are believed to have been horizontally transferred (Dagan et al. 2008). HGT in eukaryotes, is less common, its importance still being discussed but has been documented in plants (Li et al. 2014; Wickell and Li, 2020; Pereira et al. 2023), animals (Gladyshev et al. 2008; Graham et al. 2008; Moran and Jarvik, 2010), and fungi (Slot and Rokas, 2011; Mehrabi et al. 2011; Richards et al. 2011; Fitzpatrick, 2012). In fungi, HGT can have a crucial role in adaptation and, in some cases, drive significant lifestyle transitions and in some major plant pathogens affect virulence (Friesen et al. 2006; Keeling and Palmer, 2008; Fitzpatrick, 2012; McDonald et al. 2018; Zhang et al. 2019; Etten and Bhattacharya, 2020; Sahu et al. 2023; Ciach et al. 2024). Exactly how such HGTs occur in fungi is poorly understood but one avenue appears to be through horizontal chromosome transfer (HCT). HCT involves transmission of entire chromosomes from a donor to a recipient, which represents a massive HGT event potentially involving hundreds of genes located on the transmitted chromosome. In fungi, such HCTs have been reported for some accessory chromosomes, which are chromosomes that show presence/absence polymorphism. This apparent specificity of HCT for accessory chromosomes might be indicative of a regulated mechanism, possibly by the transferred accessory chromosome itself, which would be conceptually similar to conjugative bacterial plasmids, which encode the machinery for their own transfer and represent one of the pivotal avenues for HGT in prokaryotes.

## Accessory chromosomes are found in several, mostly pathogenic fungi, often affecting their pathogenicity and/or host-range

Accessory chromosomes are nonessential, linear chromosomes found in some but not all members of a population. Also known as B chromosomes, supernumerary chromosomes, dispensable chromosomes, lineage-specific chromosomes, or mini-chromosomes, they have been reported in over 3000 eukaryotic species, including plants, animals, and fungi (Houben, 2017; D’Ambrosio et al. 2017; Ferree et al. 2024). Many fungal species, particularly pathogenic fungi, possess distinct accessory chromosomes that can constitute a significant portion of their genomes. Overall, accessory chromosomes have been reported in more than 25 fungal species, including major plant pathogens like *Magnaporthe oryzae*, *Fusarium oxysporum*, *Alternaria alternata*, *F. solani* (synonym *F. vanettinii*), and *Zymoseptoria tritici* (Orbach et al. 1996; Han et al. 2001; Coleman et al. 2009; Ma et al. 2010; Goodwin et al. 2011; Langner et al. 2021). Accessory chromosomes in pathogenic fungi often carry virulence factors directly linked to pathogenicity. They can affect the host range of the fungus, for instance, the *SIX* effectors located on the accessory chromosome 14 of the plant pathogen *F. oxysporum* f.sp. *lycopersici* are required for its pathogenicity on tomato (Ma et al. 2010; Hu et al. 2012; Zhang et al. 2020). Another example are the accessory chromosomes of *A. alternata* which encode host specific toxins (Akamatsu et al. 1999; Johnson et al. 2001). Accessory chromosomes are defined by their presence/absence polymorphism, which can be affected by their segregation during mitotic and meiotic cell-divisions (Komluski et al. 2022). Readers are directed to comprehensive reviews on accessory chromosomes in fungi, which discuss their prevalence, potential function, their presence-absence polymorphism and unique characteristics (Mehrabi et al. 2011, 2017; Bertazzoni et al. 2018; Habig and Stukenbrock 2020; Witte, Villeneuve et al. 2021). We here focus on their potential for horizontal transmission – which is distinct from their vertical transmission during canonical mitosis and meiosis.

## Accessory chromosomes are horizontally transferred in some fungi

Fungal accessory chromosomes can be horizontally transferred, representing a significant horizontal gene transfer (HGT) event, often encompassing hundreds of genes and, in addition, non-genic elements. Horizontal transfer of entire accessory chromosomes has been experimentally documented in a number of fungi, including *F. oxysporum* (Ma et al. 2010; van Dam et al. 2017; Li et al. 2020), *Alternaria* spp. (Akagi et al. 2009), *Metarhizium robertsii* (Habig et al. 2024), and *Colletotrichum gloeosporioides* (He et al. 1998). In addition to experimental evidence, phylogenetic analyses provide support for horizontal transfer events of accessory chromosomes in further *F. oxysporum* species and strains (Ma et al. 2010; van Dam et al. 2017), *Alternaria* spp. (Wang et al. 2019), *M. oryzae* (Barragan et al. 2024) and between *Metarhizium* spp (Habig et al. 2024). It is important to note that HCT appears to not require meiosis and is limited to specific chromosomes (see next paragraph). This makes it distinct from (sexual) hybridzation, which is already recognized as an important factor in fungal evolution (Steensels et al. 2021).

## Reported instances of horizontal chromosome transfer (HCT) in fungi are not random but show a clear pattern of preference and specificity for accessory chromosomes

**Fig. 1:**
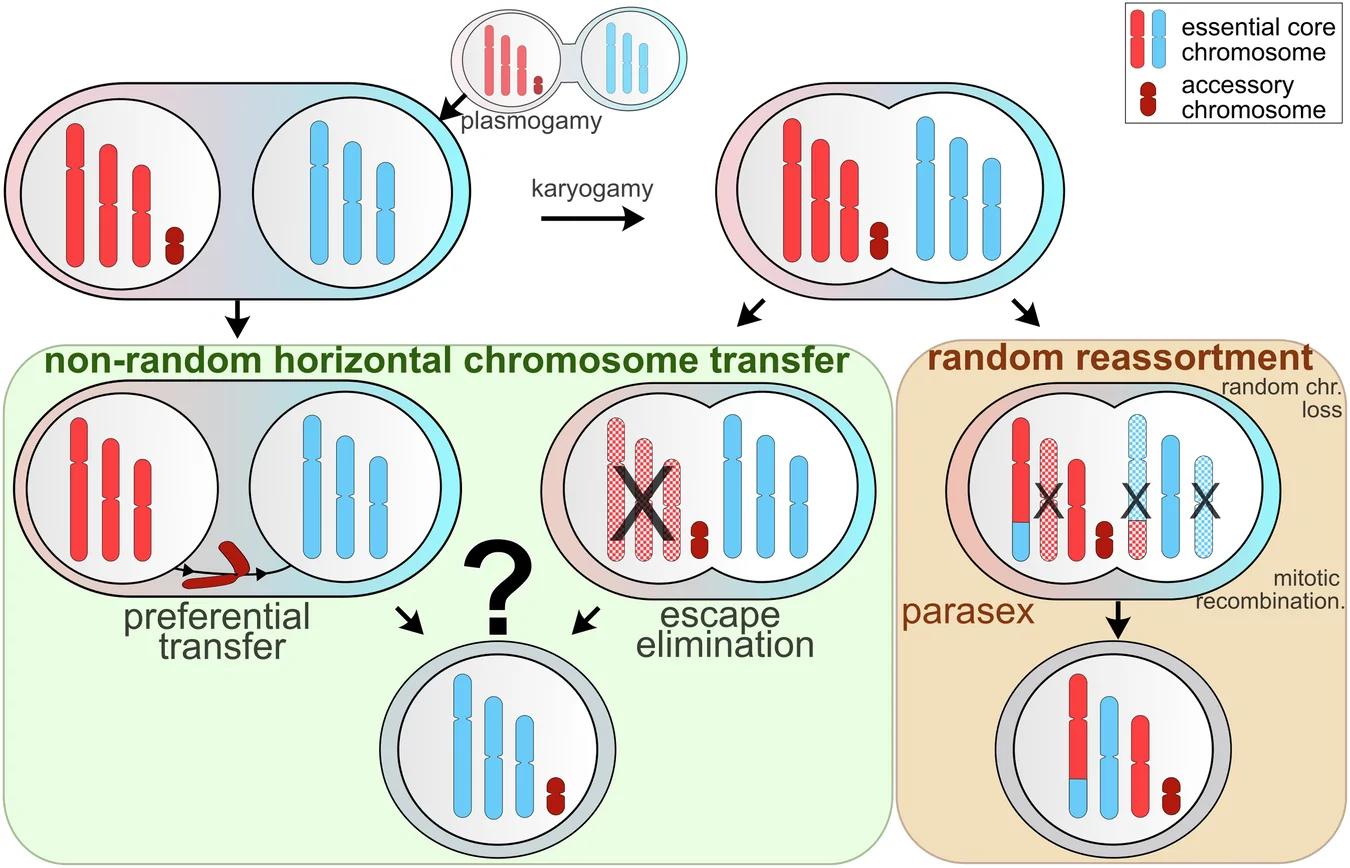
Proposed mechanism for non-random horizontal chromosome transfer, contrasted with the expected random chromosome reassortment that occur during parasexuality. First, two fungal cells or hyphae must anastomose so divergent nuclei can interact (if two haploid nuclei are involved this can lead to plasmogamy). Second, this may or may not lead to nuclear fusion (karyogamy). Left, green box: The non-random horizontal transfer of accessory chromosomes could involve preferential transfer between nuclei (or from a degraded nucleus to an intact one). Alternatively, if karyogamy occurs, there may be selective retention of the accessory chromosome while the remaining donor chromosomes are eliminated (as suggested by He et al. 1998; Vlaardingerbroek et al. 2016). Both mechanisms would require a signal that differentiates the accessory chromosome from the rest of the genome. Right, brown box: In contrast, during fungal parasexuality, following karyogamy, the diploid nucleus can revert to haploidy through subsequent, presumably random chromosome loss and in addition mitotic recombination may occur, resulting in random chromosome reassortment.

What could be a mechanism for HCT? Entire eukaryotic chromosomes are likely not stable outside cells and therefore cytoplasmic continuity - either between hyphae or germ tubes (Roca et al. 2004; Fleissner et al. 2009; Ishikawa et al. 2010) - is believed to be a prerequisite for horizontal chromosome transfer (Mehrabi et al. 2011). Following such cellular plasmogamy, heterokaryons can progress to parasexuality, a mechanism observed in some fungi that allows for the recombination of genetic material independent of meiosis (Fig. 1) (Nieuwenhuis and James 2016; Yadav et al. 2023). In this process, the ploidy of the fused nucleus (now diploid) is reduced back to haploidy through presumably random chromosome loss and possibly involving mitotic recombination. Consequently, parasexuality is considered to lead to a random reassortment of genetic material from the two fused cells. In stark contrast, none of the reported instances of fungal HCT demonstrate random reassortment of genetic material; instead, there is a marked specificity, i.e. preference for particular accessory chromosomes, even when other accessory chromosomes are also present. Furthermore, HCT very rarely involves core chromosomes. Here, we provide a brief overview of the examples of HCT in fungi, emphasizing the aspect of this specificity.

In *A. alternata*, induced protoplast fusion resulted in horizontal chromosome transfer (HCT) of an accessory chromosome, referred to as a conditionally dispensable chromosome in this fungus. Although complete genome sequencing data were not generated, there was no indication that any other genetic material was transferred from the donor to the recipient (Akagi et al. 2009). Similarly, in *C. gloeosporioides*, HCT of a 2 Mb accessory chromosome was observed between two vegetatively incompatible biotypes, involving only the accessory chromosome while no other genetic material was transferred from the donor to the recipient. Additionally, attempts to detect the transfer of core chromosomes using the same system of different fungicide resistance markers in donor and recipient strains were unsuccessful for these biotypes further indicating that the ability of horizontal transfer appears to be restricted to the accessory chromosome (He et al. 1998).

Members of the *Fusarium oxysporum* species complex (FOSC) have been most intensively studied for HCT. In this group, accessory chromosomes—referred to as lineage-specific chromosomes—are mobile and capable of horizontal transfer. Accessory chromosome 14, often called the pathogenicity chromosome, along with parts of accessory chromosomes 3 and 6, has been shown to be horizontally transferred from *F. oxysporum* f.sp. *lycopersici*, which is pathogenic on tomato, to a non-pathogenic strain, thereby rendering the recipient strain pathogenic on tomato as well. Importantly, no core chromosomes were transferred in this process (Ma et al. 2010). In a later study, again only accessory chromosome 14 was horizontally transferred, while other accessory chromosomes, albeit also being targeted and analysed similar to chromosome 14, were not transferred. In this study, parts of core chromosomes were also transmitted, but these transfers were always accompanied by the transfer of accessory chromosome 14, even though in these cases it did not carry the fungicide resistance marker that was used to detect HCT (Vlaardingerbroek et al. 2016). Similarly, in *F. oxysporum* f.sp. *radicis-cucumerinum*, an accessory chromosome was repeatedly transferred horizontally, while no other donor genetic material was transferred (van Dam et al. 2017). In summary, HCT has been frequently reported in FOSC, specifically involving one accessory chromosome, although transfers of core chromosomal material can occur in rare cases.

Very recently, horizontal chromosome transfer (HCT) of accessory chromosomes has been reported in the entomopathogenic *Metarhizium robertsii* and in *Magnaporthe* (syn. *Pyricularia*) *oryzae*. In *M robertsii*, an accessory chromosome (termed chrA) was frequently horizontally transmitted from a donor to a recipient strain, while the transfer of any other genetic material could be ruled out with high certainty based on long- and short-read sequencing data (Habig et al. 2024). This transfer occurred during co-infections of a host insect by different strains of *M. robertsii*, and apparently affected the fitness of the recipient fungus. Notably, additional phylogenetic analysis showed that chrA had also been horizontally transmitted between distinct *Metarhizium* species. In addition, in *M. oryzae*, a plant pathogen responsible for crop pandemics through asexual reproduction of clonal lineages, accessory chromosomes (referred to as mini-chromosomes in this fungus) contain virulence-related genes and have been horizontally transferred. In particular, the accessory chromosome mChrA—a 1.2 Mb mini-chromosome containing 191 genes—has been frequently horizontally transferred between clonal lineages of *M. oryzae* in the recent past. Again, notably, there was no indication of core genome exchange (Langner et al. 2021; Barragan et al. 2024).

In summary, all reported horizontal chromosome transfers showed a high degree of propensity and specificity towards involving a specific accessory chromosome and hence cannot be solely explained by the assumingly random processes of parasexuality (Fig. 1).

## The non-random process of horizontal chromosome transfer (HCT) requires a distinguishing signal that differentiates mobile chromosomes from the rest of the genome

The non-random nature of HCT has already been previously acknowledged, and two primary mechanisms causing this specificity have been proposed (He et al. 1998; Vlaardingerbroek et al. 2016). The first suggests that the mobile chromosome is preferentially transferred during the heterokaryon stage from one (potentially degrading) donor nucleus to the recipient nucleus (Fig. 1). The second mechanism posits that following karyogamy, the mobile chromosome avoids a targeted genome elimination process (Fig. 1). A related phenomenon to the former has been observed in the budding yeast *Saccharomyces cerevisiae*, where a mutant with defective nuclear fusion temporarily creates heterokaryons, which facilitate chromosome transfer between nuclei before one of them is lost – a process termed chromoduction (Conde and Fink 1976; Dutcher 1981; Ji et al. 1993; Zhao et al. 2022). Similarly, a process akin to the second mechanism—where all but the mobile chromosomes are degraded—occurs during programmed DNA elimination observed in various plants and animals (Dedukh and Krasikova, 2022). Both mechanisms require a signal that distinguishes mobile chromosomes from the rest of the genome, either for preferential transfer or to avoid elimination. We speculate such a signal could be epigenetic and we base our speculation on previous examples of histone modification-regulated genome elimination, such as in the jewel wasp *Nasonia vitripennis* (Aldrich et al. 2017; Lee et al. 2023), and the distinct epigenetic modifications that differentiate fungal accessory chromosomes from core chromosomes (Schotanus et al. 2015; Freitag, 2017; Habig et al. 2021). An alternative scenario for the observed propensity of HCT towards accessory chromosomes could be that accessory chromosomes are usually smaller and contain a lower number of genes. Hence, these chromosomes might face a lower probability of incompatibilities with the recipient genomes compared to larger and more gene-rich core chromosomes. Therefore, we would only see HCT of accessory chromosomes because HCT of other chromosomes might be lethal. However, this scenario requires lethal or highly disadvantageous interactions for every core chromosome, which we consider unlikely – also because small core chromosomes can be similar or even smaller in size to accessory chromosomes, e.g., in *F. oxysporum* (Ma et al. 2010). Therefore, we do not consider the size of or the number of genes on accessory chromosomes to be the factor that results in the observed propensity of HCT towards accessory chromosomes.

## Could mobile accessory chromosomes encode the functions for their own horizontal transfer?

Functional annotation of genes on the mobile accessory chromosomes chrA and chrB in *M. robertsii* revealed an enrichment of genes that potentially influence chromatin conformation (Habig et al. 2024). Notably, chrA harbors two putative histone H2B genes, two putative histone lysine N-methyltransferases (H3 lysine-4 specific) (Freitag, 2017), and a putative histone acetyltransferase similar to GCN5 (predominantly H3 lysine 14 specific) (Li and Shogren-Knaak, 2009). Importantly, these genes are not paralogs of their canonical counterparts on core chromosomes but true xenologs, suggesting they may exhibit different specificities. Similarly, the mobile accessory chromosome mChrA in *M. oryzae* is enriched (among others) for genes involved in the GO-terms for the molecular function of chromatin binding and for genes encoding products located in the nucleosome (Barragan et al. 2024). Moreover, mChrA of *M. oryzae* also contains genes encoding two putative histones (g11257 and g11315) (Barragan et al. 2024). The presence of these potentially chromatin-affecting genes on the mobile accessory chromosome chrA in *M. robertsii* as well as the mobile accessory chromosome mchrA in *M. oryzae* raises the intriguing possibility that these mobile chromosomes encode functions necessary for their own preferential transfer, conceptually similar to conjugative plasmids in bacteria, which also encode the machinery for their own horizontal transfer. Given that HCT of fungal accessory chromosomes in other species also display some degree of specificity, these mobile chromosomes could potentially also be involved in their own transmission as well. For example, a secondary metabolite cluster for the production of apicidin, a histone deacetylase inhibitor, is located on an accessory chromosome in *F. poae* (Witte et al. 2021) and could be involved in generating the chromatin conformation signal that distinguishes the accessory chromosomes from the core chromosomes. However, to date, genes located on fungal accessory chromosomes are poorly annotated functionally, with few characterized beyond putative effectors. For instance, in *A. alternata*, more than 25 genes identified on an accessory chromosome of multiple isolates lack any functional annotation, and most appear to be unique to the *Alternaria* genus. (Harimoto et al. 2007; Gai et al. 2021). Functionally annotating these and other genes on accessory chromosomes could be highly insightful, potentially uncovering functions that, for example, influence chromatin conformation. These would be intriguing targets for testing their impact on the horizontal transfer rates of these chromosomes.

## How widespread is horizontal chromosome transfer of accessory chromosomes?

**Fig. 2:**
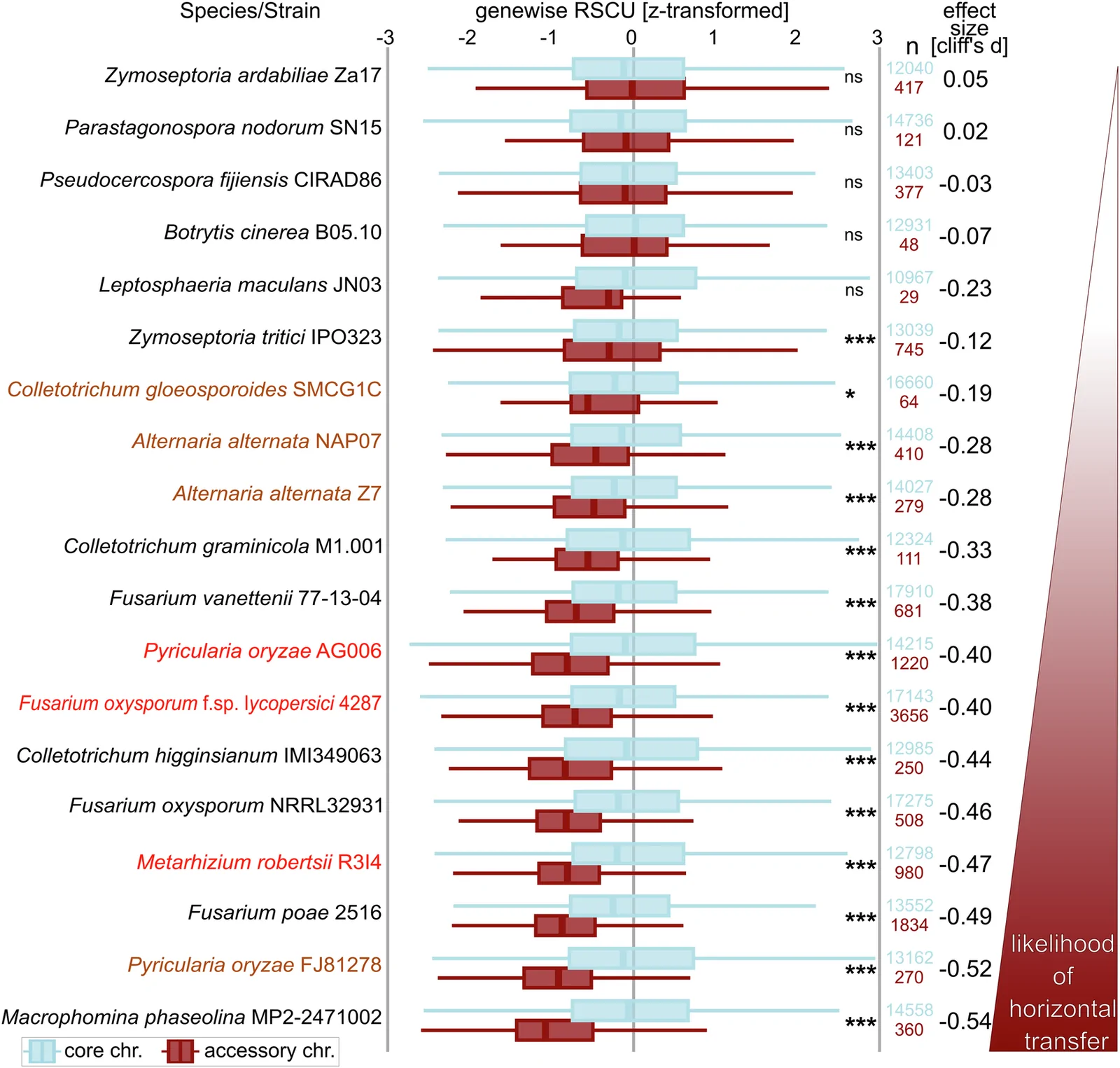
Differences in codon usage bias between core and accessory chromosomes may indicate horizontal transfer. Codon usage bias, assessed through gene-wise RSCU (Steenwyk et al. 2022) comparisons between core (blue) and accessory chromosomes (dark red), z-transformed for easier visual comparison (see also Fig. S1 for untransformed data). Fungal strains with documented horizontal transfer are highlighted in red, while those with evidence of transfer in different strains of the same species are marked in orange. Species are organized according to increasing effect sizes, represented by the absolute value of Cliff’s d, for those exhibiting significant differences. Statistical significance was determined using the Wilcoxon rank sum test (with Hochberg adjustment for multiple testing). Not significant (ns), *p < 0.05, **p < 0.005, ***p < 0.0005. *n* represents the number of cds that were included for core (blue) and accessory chromosomes (red).

Despite the presence of accessory chromosomes in more than 25 fungal species (Bertazzoni et al. 2018; Komluski et al. 2022), horizontal transfer has only been reported in five species. Since HCT appears to specifically involve accessory chromosomes, those accessory chromosomes, for which HCT have not been reported yet, might still have experienced HCTs in the past and the consequences of these transfers might still be detectable. One measurable indication of past horizontal transfers is variation in the relative synonymous codon usage (RSCU) between core and accessory chromosomes. RSCU, introduced by Sharp and Li (Sharp and Li, 1986), measures codon usage bias, which can be influenced by mutational processes such as GC content and nucleotide sequence context, as seen in mechanisms like Repeat-Induced Point (RIP) mutation (Galagan and Selker, 2004; Hershberg and Petrov, 2008; Plotkin and Kudla, 2011). Crucially, codon usage bias is also shaped by selective processes, where certain preferred codons are translated more accurately or efficiently (Hershberg and Petrov, 2008). This form of translational selection is thought to correlate with relative tRNA abundance (Plotkin and Kudla, 2011). Thus, both mutational and selective pressures may vary between species and genomes, potentially leading to differences in codon usage bias. Since horizontal chromosome transfer (HCT) involves the movement of an accessory chromosome from one genomic background—where it was shaped by species-specific mutational and selective forces—into a new genomic background where these forces may differ, we hypothesize that HCT might be reflected in differences in the codon usage bias between core and accessory chromosomes (Fig. 2). Indeed, differences in codon usage bias between core and accessory chromosomes, had previously been reported for several fungal species, e.g. *F. oxysporum*, *F. vanettinii* MPVI, *A. arborescens, C. graminicola* and *M. robertsii* (Coleman et al. 2009; Ma et al. 2010; Goodwin et al. 2011; Hu et al. 2012; Becerra et al. 2023; Habig et al. 2024).

However, it is important to note that differences in codon usage bias may also arise from mutational or selective processes that affect accessory chromosomes differently than core chromosomes independently of horizontal transfer. For instance, accessory chromosomes may exhibit higher mutation rates due to distinct histone modifications (Habig et al. 2021), or in sexually reproducing organisms, these chromosomes may be subjected to less efficient selection due to their smaller effective population sizes. Additionally, for individual genes, codon usage bias is not a sensitive indicator of past horizontal transfer (Koski et al. 2001). Nevertheless, because HCT typically involves the transfer of a large number of genes and non-genic elements, we believe that analysing codon usage bias across all transcripts from core and accessory chromosomes could reveal significant differences that warrant further investigation. It is also important to note that the absence of differences in codon usage bias does not necessarily rule out past horizontal transfers, as HCT could occur between species with similar genome-wide codon usage bias. Therefore, while a pronounced difference in codon usage bias may be a strong indicator of past horizontal transfer, the lack of such differences should not be interpreted as definitive evidence against it. To explore this hypothesis, we analysed codon usage differences between core and accessory chromosomes in fungal species where HCT has been experimentally or phylogenetically demonstrated (Fig. 2). Additionally, we included fungal species with available genome sequencing data that distinguish between core and accessory chromosomes, to explore how widespread signals of potential HCT are among fungal accessory chromosomes. To be able to compare the potential differences between species and strains we annotated the genes ab initio for all (see Text S1 for Methods and Materials).

## Horizontally transferred accessory chromosomes exhibit distinct codon usage biases, which is a characteristic shared by many other accessory chromosomes and suggests more widespread horizontal chromosome transfers

By comparing codon usage bias between core and accessory chromosomes across 19 strains from 16 distinct fungal species (see Text S1 for details), we found that five species—*Parastagonospora nodorum*, *Botrytis cinerea*, *Pseudocercospora fijiensis*, and *Leptosphaeria maculans*—showed no significant difference in codon usage between core and accessory chromosomes (Fig. 2). Notably, these species are known or assumed to undergo frequent sexual reproduction (Rouxel and Balesdent, 2005; Williamson et al. 2007; Churchill, 2010; Stukenbrock et al. 2012; Gao et al. 2016), and hence the accessory chromosomes might be primarily vertically inherited. Conversely, we found significant and highly pronounced differences (measured by the effect size using Cliff’s delta, a robust non-parametric estimate of effect sizes that does not assume normally distributed data) between core chromosomes, and accessory chromosomes, for which horizontal transfer had been experimentally or phylogenetically demonstrated. This included *F. oxysporum* f.sp. *lycopersici* strain 4287 (Ma et al. 2010), *M. oryzae* strain AG006 (Barragan et al. 2024), and *M. robertsii* strain R3I4 (Habig et al. 2024) (see Fig. 2 in red). Additionally, significant differences were also detected in strains of species, for which horizontal transfer was experimentally shown in only a different strain than that with sequencing data, including *C. gloeosporioides* (He et al. 1998) and *A. alternata* (Akagi et al. 2009) (see Fig. 2, orange). Hence, significant and pronounced differences in the RSCU were detectable for all accessory chromosomes for which horizontal transmission was previously shown. For the remaining accessory chromosomes, for which it is unknown whether horizontal transfer occurs, we see a variation from relatively small but significant differences in *Z. tritici* IPO323 (Cliff’s *d* = −0.11), to large significant difference in *Macrophomina phaesolina* strain MP2-2471002 (Cliff’s *d* = −0.57). In particular the large, significant difference in codon usage bias for the accessory chromosome(s) of *M. phaseolina*, a broad host-range asexual plant pathogen for which parasexuality is suspected (Jones et al. 1998; Marquez et al. 2021), as well as *F. poae* and *C. higgensianum* is striking and should warrant further investigation into HCT. In general, the fact that the majority of accessory chromosomes show a large significant difference in codon usage could be indicative of many more horizontal chromosome transfers of accessory chromosomes than are currently known.

### Final remarks

Horizontal chromosome transfer (HCT) has been experimentally and phylogenetically demonstrated in a handful fungal species with accessory chromosomes, often significantly impacting the fitness, pathogenicity, or life-history traits of the recipient fungus. The fact that such HCTs consistently show specificity for accessory chromosomes suggests that it may be a regulated process, potentially enabling the extensive horizontal transfer of both genic and non-genic elements and thereby influencing fungal genome evolution. A simple comparison of relative synonymous codon usage (RCSU) bias between accessory and core chromosomes suggests that HCT might be much more prevalent among fungal accessory chromosomes than previously thought, highlighting the potential significance of this process in fungal evolution. Mobile accessory chromosomes, with distinct transmission systems compared to core chromosomes, may exhibit separate evolutionary dynamics and potentially act as selfish genetic elements. Unraveling the signals and genetic mechanisms underlying HCT will require a functional dissection of genes located on accessory chromosomes, especially genes that might influence chromatin conformation or chromosome segregation. This focus could be particularly valuable for understanding the unique and impactful phenomenon of horizontal transfer of entire chromosomes.

## Supplementary information

Figure S1

Table S1

Table S2

Table S3

Supporting Information

## References

[CR1] Akagi Y, Taga M, Yamamoto M, Tsuge T, Fukumasa-Nakai Y, Otani H et al. (2009) Chromosome constitution of hybrid strains constructed by protoplast fusion between the tomato and strawberry pathotypes of Alternaria alternata. J Gen Plant Pathol 75:101

[CR2] Akamatsu H, Taga M, Kodama M, Johnson R, Otani H, Kohmoto K (1999) Molecular karyotypes for Alternaria plant pathogens known to produce host-specific toxins. Curr Genet 35:647–656 10467010 10.1007/s002940050464

[CR3] Aldrich JC, Leibholz A, Cheema MS, Ausiό J, Ferree PM (2017) A ‘selfish’ B chromosome induces genome elimination by disrupting the histone code in the jewel wasp Nasonia vitripennis. Sci Rep. 7:42551 28211924 10.1038/srep42551 PMC5304203

[CR4] Barragan AC, Latorre SM, Malmgren A, Harant A, Win J, Sugihara Y et al. (2024) Multiple horizontal mini-chromosome transfers drive genome evolution of clonal blast fungus lineages. Mol Biol Evol 41:msae164 39107250 10.1093/molbev/msae164 PMC11346369

[CR5] Becerra S, Baroncelli R, Boufleur TR, Sukno SA, Thon MR (2023) Chromosome-level analysis of the Colletotrichum graminicola genome reveals the unique characteristics of core and minichromosomes. Front Microbiol 14:129319 10.3389/fmicb.2023.1129319 PMC10076810 37032845

[CR6] Bertazzoni S, Williams AH, Jones DA, Syme RA, Tan K-C, Hane JK (2018) Accessories make the outfit: accessory chromosomes and other dispensable DNA regions in plant-pathogenic fungi. MPMI 31:779–788 29664319 10.1094/MPMI-06-17-0135-FI

[CR7] Churchill ACL (2010) Mycosphaerella fijiensis, the black leaf streak pathogen of banana: progress towards understanding pathogen biology and detection, disease development, and the challenges of control. Mol Plant Pathol 12:307 21453427 10.1111/j.1364-3703.2010.00672.x PMC6640443

[CR8] Ciach MA, Pawłowska J, Górecki P, Muszewska A (2024) The interkingdom horizontal gene transfer in 44 early diverging fungi boosted their metabolic, adaptive, and immune capabilities. Evol Lett, qrae009:526–538 10.1093/evlett/qrae009 PMC11291939 39100235

[CR9] Coleman JJ, Rounsley SD, Rodriguez-Carres M, Kuo A, Wasmann CC, Grimwood J et al. (2009) The genome of nectria haematococca: contribution of supernumerary chromosomes to gene expansion. PLOS Genet 5:e1000618 19714214 10.1371/journal.pgen.1000618 PMC2725324

[CR10] Conde J, Fink GR (1976) A mutant of Saccharomyces cerevisiae defective for nuclear fusion. Proc Natl Acad Sci 73:3651–3655 790391 10.1073/pnas.73.10.3651 PMC431176

[CR11] Dagan T, Artzy-Randrup Y, Martin W (2008) Modular networks and cumulative impact of lateral transfer in prokaryote genome evolution. Proc Natl Acad Sci 105:10039–10044 18632554 10.1073/pnas.0800679105 PMC2474566

[CR12] D’Ambrosio U, Alonso‐Lifante MP, Barros K, Kovařík A, Mas De Xaxars G, Garcia S (2017) B‐chrom: a database on B‐chromosomes of plants, animals and fungi. N Phytol 216:635–642 10.1111/nph.14723 28742254

[CR13] Dedukh D, Krasikova A (2022) Delete and survive: strategies of programmed genetic material elimination in eukaryotes. Biol Rev 97:195–216 34542224 10.1111/brv.12796 PMC9292451

[CR14] Dutcher SK (1981) Internuclear transfer of genetic information in kar1-1/KAR1 heterokaryons in saccharomyces cerevisiae. Mol Cell Biol 1:245–253 6765600 10.1128/mcb.1.3.245-253.1981 PMC369668

[CR15] Etten JV, Bhattacharya D (2020) Horizontal gene transfer in eukaryotes: Not if, but How Much? Trends Genet 36:915–925 33012528 10.1016/j.tig.2020.08.006

[CR16] Ferree PM, Blagojević J, Houben A, Martins C, Trifonov VA, Vujošević M (2024) What is a B chromosome? Early definitions revisited. G3 Genes|Genomes|Genet 14:jkae068 38626314 10.1093/g3journal/jkae068 PMC11152065

[CR17] Fitzpatrick DA (2012) Horizontal gene transfer in fungi. FEMS Microbiol Lett 329:1–8 22112233 10.1111/j.1574-6968.2011.02465.x

[CR18] Fleissner A, Leeder AC, Roca MG, Read ND, Glass NL (2009) Oscillatory recruitment of signaling proteins to cell tips promotes coordinated behavior during cell fusion. Proc Natl Acad Sci USA 106:19387–19392 19884508 10.1073/pnas.0907039106 PMC2780775

[CR19] Freitag M (2017) Histone methylation by SET domain proteins in fungi. Annu Rev Microbiol 71:413–439 28715960 10.1146/annurev-micro-102215-095757

[CR20] Friesen TL, Stukenbrock EH, Liu Z, Meinhardt S, Ling H, Faris JD et al. (2006) Emergence of a new disease as a result of interspecific virulence gene transfer. Nat Genet 38:953–956 16832356 10.1038/ng1839

[CR21] Gai Y, Ma H, Chen Y, Li L, Cao Y, Wang M et al. (2021) Chromosome-scale genome sequence of alternaria alternata causing alternaria brown spot of citrus. MPMI 34:726–732 33689393 10.1094/MPMI-10-20-0278-SC

[CR22] Galagan JE, Selker EU (2004) RIP: the evolutionary cost of genome defense. Trends Genet 20:417–423 15313550 10.1016/j.tig.2004.07.007

[CR23] Gao Y, Liu Z, Faris JD, Richards J, Brueggeman RS, Li X et al. (2016) Validation of genome-wide association studies as a tool to identify virulence factors in parastagonospora nodorum. Phytopathol® 106:1177–1185 10.1094/PHYTO-02-16-0113-FI 27442533

[CR24] Gladyshev EA, Meselson M, Arkhipova IR (2008) Massive horizontal gene transfer in bdelloid rotifers. Science 320:1210–1213 18511688 10.1126/science.1156407

[CR25] Goodwin SB, M’Barek SB, Dhillon B, Wittenberg AHJ, Crane CF, Hane JK et al. (2011) Finished genome of the fungal wheat pathogen mycosphaerella graminicola reveals dispensome structure, chromosome plasticity, and stealth pathogenesis. PLoS Genet 7:e1002070 21695235 10.1371/journal.pgen.1002070 PMC3111534

[CR26] Graham LA, Lougheed SC, Ewart KV, Davies PL (2008) Lateral transfer of a lectin-like antifreeze protein gene in fishes. PLoS ONE 3:e2616 18612417 10.1371/journal.pone.0002616 PMC2440524

[CR27] Habig M, Grasse AV, Müller J, Stukenbrock EH, Leitner H, Cremer S (2024) Frequent horizontal chromosome transfer between asexual fungal insect pathogens. Proc Natl Acad Sci USA 121:e2316284121 38442176 10.1073/pnas.2316284121 PMC10945790

[CR28] Habig M, Lorrain C, Feurtey A, Komluski J, Stukenbrock EH (2021) Epigenetic modifications affect the rate of spontaneous mutations in a pathogenic fungus. Nat Commun 12:5869 34620872 10.1038/s41467-021-26108-y PMC8497519

[CR29] Habig M, Stukenbrock EH (2020). Origin, Function, and Transmission of Accessory Chromosomes. In: Benz JP, Schipper K (eds) *Genetics and Biotechnology*, The Mycota. Springer International Publishing: Cham, pp 25–47

[CR30] Han Y, Liu X, Benny U, Kistler HC, VanEtten HD (2001) Genes determining pathogenicity to pea are clustered on a supernumerary chromosome in the fungal plant pathogen Nectria haematococca. Plant J 25:305–314 11208022 10.1046/j.1365-313x.2001.00969.x

[CR31] Harimoto Y, Hatta R, Kodama M, Yamamoto M, Otani H, Tsuge T (2007) Expression profiles of genes encoded by the supernumerary chromosome controlling am-toxin biosynthesis and pathogenicity in the apple pathotype of alternaria alternata. MPMI 20:1463–1476 17990954 10.1094/MPMI-20-12-1463

[CR32] He C, Rusu AG, Poplawski AM, Irwin JAG, Manners JM (1998) Transfer of a supernumerary chromosome between vegetatively incompatible biotypes of the fungus colletotrichum gloeosporioides. Genetics 150:1459–1466 9832523 10.1093/genetics/150.4.1459 PMC1460434

[CR33] Hershberg R, Petrov DA (2008) Selection on codon bias. Annu Rev Genet 42:287–299 18983258 10.1146/annurev.genet.42.110807.091442

[CR34] Houben A (2017) B Chromosomes – a matter of chromosome drive. Front Plant Sci 8:210 10.3389/fpls.2017.00210 PMC5309253 28261259

[CR35] Hu J, Chen C, Peever T, Dang H, Lawrence C, Mitchell T (2012) Genomic characterization of the conditionally dispensable chromosome in Alternaria arborescens provides evidence for horizontal gene transfer. BMC Genom 13:171 10.1186/1471-2164-13-171 PMC3443068 22559316

[CR36] Ishikawa FH, Souza EA, Read ND, Roca MG (2010) Live-cell imaging of conidial fusion in the bean pathogen, *Colletotrichum lindemuthianum*. Fungal Biol 114:2–9 20965055 10.1016/j.funbio.2009.11.006

[CR37] Ji H, Moore DP, Blomberg MA, Braiterman LT, Voytas DF, Natsoulis G et al. (1993) Hotspots for unselected Ty1 transposition events on yeast chromosome III are near tRNA genes and LTR sequences. Cell 73:1007–1018 8388781 10.1016/0092-8674(93)90278-x

[CR38] Johnson LJ, Johnson RD, Akamatsu H, Salamiah A, Otani H, Kohmoto K et al. (2001) Spontaneous loss of a conditionally dispensable chromosome from the Alternaria alternata apple pathotype leads to loss of toxin production and pathogenicity. Curr Genet 40:65–72 11570518 10.1007/s002940100233

[CR39] Jones RW, Canada S, Wang H (1998) Highly variable minichromosomes and highly conserved endoglucanase genes in the phytopathogenic fungus Macrophomina phaseolina. Can J Bot 76:694–698

[CR40] Keeling PJ, Palmer JD (2008) Horizontal gene transfer in eukaryotic evolution. Nat Rev Genet 9:605–618 18591983 10.1038/nrg2386

[CR41] Komluski J, Stukenbrock EH, Habig M (2022) Non-Mendelian transmission of accessory chromosomes in fungi. Chromosome Res 30:241–253 35881207 10.1007/s10577-022-09691-8 PMC9508043

[CR42] Koski LB, Morton RA, Golding GB (2001) Codon bias and base composition are poor indicators of horizontally transferred genes. Mol Biol Evolution 18:404–412 10.1093/oxfordjournals.molbev.a003816 11230541

[CR43] Langner T, Harant A, Gomez-Luciano LB, Shrestha RK, Malmgren A, Latorre SM et al. (2021) Genomic rearrangements generate hypervariable mini-chromosomes in host-specific isolates of the blast fungus. PLoS Genet 17:e1009386 33591993 10.1371/journal.pgen.1009386 PMC7909708

[CR44] Lee H, Seo P, Teklay S, Yuguchi E, Benetta ED, Werren JH et al. (2023) Ability of a selfish B chromosome to evade genome elimination in the jewel wasp, Nasonia vitripennis. Heredity 131:230–237 37524915 10.1038/s41437-023-00639-0 PMC10462710

[CR45] Li J, Fokkens L, Conneely LJ, Rep M (2020) Partial pathogenicity chromosomes in Fusarium oxysporum are sufficient to cause disease and can be horizontally transferred. Environ Microbiol 22:4985–5004 32452643 10.1111/1462-2920.15095 PMC7818268

[CR46] Li S, Shogren-Knaak MA (2009) The Gcn5 bromodomain of the SAGA complex facilitates cooperative and cross-tail acetylation of nucleosomes*. J Biol Chem 284:9411–9417 19218239 10.1074/jbc.M809617200 PMC2666593

[CR47] Li F-W, Villarreal JC, Kelly S, Rothfels CJ, Melkonian M, Frangedakis E et al. (2014) Horizontal transfer of an adaptive chimeric photoreceptor from bryophytes to ferns. Proc Natl Acad Sci 111:6672–6677 24733898 10.1073/pnas.1319929111 PMC4020063

[CR48] Ma L-J, van der Does HC, Borkovich KA, Coleman JJ, Daboussi M-J, Di Pietro A et al. (2010) Comparative genomics reveals mobile pathogenicity chromosomes in fusarium. Nature 464:367–373 20237561 10.1038/nature08850 PMC3048781

[CR49] Marquez N, Giachero ML, Declerck S, Ducasse DA (2021) Macrophomina phaseolina: General Characteristics of Pathogenicity and Methods of Control. Front Plant Sci 12:634397 33968098 10.3389/fpls.2021.634397 PMC8100579

[CR50] McDonald MC, Ahren D, Simpfendorfer S, Milgate A, Solomon PS (2018) The discovery of the virulence gene ToxA in the wheat and barley pathogen Bipolaris sorokiniana. Mol Plant Pathol 19:432–439 28093843 10.1111/mpp.12535 PMC6638140

[CR51] Mehrabi R, Bahkali AH, Abd-Elsalam KA, Moslem M, Ben M’Barek S, Gohari AM et al. (2011) Horizontal gene and chromosome transfer in plant pathogenic fungi affecting host range. FEMS Microbiol Rev 35:542–554 21223323 10.1111/j.1574-6976.2010.00263.x

[CR52] Mehrabi R, Mirzadi Gohari A, Kema GHJ (2017) Karyotype variability in plant-pathogenic fungi. Annu Rev Phytopathol 55:483–503 28777924 10.1146/annurev-phyto-080615-095928

[CR53] Moran NA, Jarvik T (2010) Lateral transfer of genes from fungi underlies carotenoid production in aphids. Science 328:624–627 20431015 10.1126/science.1187113

[CR54] Nieuwenhuis BPS, James TY (2016) The frequency of sex in fungi. Philos Trans R Soc B Biol Sci 371:20150540 10.1098/rstb.2015.0540 PMC5031624 27619703

[CR55] Orbach MJ, Chumley FG, Valent B (1996) Electrophoretic karyotypes of Magnaporthe grisea pathogens of diverse grasses. Mol Plant-Microbe Interact 9:261–271

[CR56] Pereira L, Christin P-A, Dunning LT (2023) The mechanisms underpinning lateral gene transfer between grasses. Plants, People, Planet 5:672–682

[CR57] Plotkin JB, Kudla G (2011) Synonymous but not the same: the causes and consequences of codon bias. Nat Rev Genet 12:32–42 21102527 10.1038/nrg2899 PMC3074964

[CR58] Richards TA, Leonard G, Soanes DM, Talbot NJ (2011) Gene transfer into the fungi. Fungal Biol Rev 25:98–110

[CR59] Roca MG, Davide LC, Davide LMC, Mendes-Costa MC, Schwan RF, Wheals AE (2004) Conidial anastomosis fusion between Colletotrichum species. Mycol Res 108:1320–1326 15587065 10.1017/s0953756204000838

[CR60] Rouxel T, Balesdent MH (2005) The stem canker (blackleg) fungus, Leptosphaeria maculans, enters the genomic era. Mol Plant Pathol 6:225–241 20565653 10.1111/j.1364-3703.2005.00282.x

[CR61] Sahu N, Indic B, Wong-Bajracharya J, Merényi Z, Ke H-M, Ahrendt S et al. (2023) Vertical and horizontal gene transfer shaped plant colonization and biomass degradation in the fungal genus Armillaria. Nat Microbiol 8:1668–1681 37550506 10.1038/s41564-023-01448-1 PMC7615209

[CR62] Schotanus K, Soyer JL, Connolly LR, Grandaubert J, Happel P, Smith KM et al. (2015) Histone modifications rather than the novel regional centromeres of Zymoseptoria tritici distinguish core and accessory chromosomes. Epigenetics Chromatin 8:41 26430472 10.1186/s13072-015-0033-5 PMC4589918

[CR63] Sharp PM, Li W-H (1986) An evolutionary perspective on synonymous codon usage in unicellular organisms. J Mol Evol 24:28–38 3104616 10.1007/BF02099948

[CR64] Slot JC, Rokas A (2011) Horizontal transfer of a large and highly toxic secondary metabolic gene cluster between fungi. Curr Biol 21:134–139 21194949 10.1016/j.cub.2010.12.020

[CR65] Steensels J, Gallone B, Verstrepen KJ (2021) Interspecific hybridization as a driver of fungal evolution and adaptation. Nat Rev Microbiol 19:485–500 33767366 10.1038/s41579-021-00537-4

[CR66] Steenwyk JL, Buida III TJ, Gonçalves C, Goltz DC, Morales G, Mead ME et al. (2022) BioKIT: a versatile toolkit for processing and analyzing diverse types of sequence data. Genetics 221:iyac079 35536198 10.1093/genetics/iyac079 PMC9252278

[CR67] Stukenbrock EH, Quaedvlieg W, Javan-Nikhah M, Zala M, Crous PW, McDonald BA (2012) Zymoseptoria ardabiliae and Z. pseudotritici, two progenitor species of the septoria tritici leaf blotch fungus Z. tritici (synonym: Mycosphaerella graminicola). Mycologia 104:1397–1407 22675045 10.3852/11-374

[CR68] van Dam P, Fokkens L, Ayukawa Y, van der Gragt M, ter, Horst A, Brankovics B et al. (2017) A mobile pathogenicity chromosome in Fusarium oxysporum for infection of multiple cucurbit species. Sci Rep. 7:9042 28831051 10.1038/s41598-017-07995-y PMC5567276

[CR69] Vlaardingerbroek I, Beerens B, Rose L, Fokkens L, Cornelissen BJC, Rep M (2016) Exchange of core chromosomes and horizontal transfer of lineage-specific chromosomes in Fusarium oxysporum. Environ Microbiol 18:3702–3713 26941045 10.1111/1462-2920.13281

[CR70] Wang M, Fu H, Shen X-X, Ruan R, Rokas A, Li H (2019) Genomic features and evolution of the conditionally dispensable chromosome in the tangerine pathotype of Alternaria alternata. Mol Plant Pathol 20:1425–1438 31297970 10.1111/mpp.12848 PMC6792136

[CR71] Wickell DA, Li F-W (2020) On the evolutionary significance of horizontal gene transfers in plants. N Phytol 225:113–117 10.1111/nph.16022 31347197

[CR72] Williamson B, Tudzynski B, Tudzynski P, Van Kan JAL (2007) Botrytis cinerea: the cause of grey mould disease. Mol Plant Pathol 8:561–580 20507522 10.1111/j.1364-3703.2007.00417.x

[CR73] Witte TE, Harris LJ, Nguyen HDT, Hermans A, Johnston A, Sproule A et al. (2021) Apicidin biosynthesis is linked to accessory chromosomes in Fusarium poae isolates. BMC Genom 22:591 10.1186/s12864-021-07617-y PMC8340494 34348672

[CR74] Witte TE, Villeneuve N, Boddy CN, Overy DP (2021) Accessory chromosome-acquired secondary metabolism in plant pathogenic fungi: the evolution of biotrophs into host-specific pathogens. Front Microbiol 12:700 10.3389/fmicb.2021.664276 PMC8102738 33968000

[CR75] Yadav V, Sun S, Heitman J (2023) On the evolution of variation in sexual reproduction through the prism of eukaryotic microbes. Proc Natl Acad Sci 120:e2219120120 36867686 10.1073/pnas.2219120120 PMC10013875

[CR76] Zhang Q, Chen X, Xu C, Zhao H, Zhang X, Zeng G et al. (2019) Horizontal gene transfer allowed the emergence of broad host range entomopathogens. Proc Natl Acad Sci 116:7982–7989 30948646 10.1073/pnas.1816430116 PMC6475382

[CR77] Zhang Y, Yang H, Turra D, Zhou S, Ayhan DH, DeIulio GA et al. (2020) The genome of opportunistic fungal pathogen Fusarium oxysporum carries a unique set of lineage-specific chromosomes. Commun Biol 3:50 32005944 10.1038/s42003-020-0770-2 PMC6994591

[CR78] Zhao Y, Coelho C, Hughes AL, Lazar-Stefanita L, Yang S, Brooks AN, et al. (2022). Debugging and consolidating multiple synthetic chromosomes reveals combinatorial genetic interactions: 2022.04.11.486913 10.1016/j.cell.2023.09.025 37944511

