# Supplementary material for: Horizontal transfer of accessory chromosomes in fungi – a regulated process for exchange of genetic material?
Source: Heredity (Edinb). 2025 Feb 10;135(8):590–6. doi: 10.1038/s41437-025-00746-0 (PMC13545237; doi:10.1038/s41437-025-00746-0)
Supplement: Supplementary file 1 [file 41437_2025_746_MOESM5_ESM.docx]

**Supporting Information for**

Horizontal transfer of accessory chromosomes in fungi – a regulated process for exchange of genetic material?

*Authors:*

*Michael Habig^1^*, Satish Kumar Patneedi^1,2^, Remco Stam^2^, Henrik Hjarvard De Fine Licht^3^*

*Author affiliation:*

*1) Fungal Evolutionary Genetics, Christian-Albrechts University of Kiel, Germany,*

*2) Department of Phytopathology and Crop protection, Christian-Albrechts University of Kiel, Germany,*

*3) Section for Organismal Biology, Department of Plant and Environmental Sciences, University of Copenhagen, Denmark*

*Corresponding author:*

**Michael Habig, email: mhabig@bot.uni-kiel.de*Habig, email: [mhabig@bot.uni-kiel.de](mailto:mhabig@bot.uni-kiel.de)

**This PDF file includes:**

Supporting text S1

SI References

# Content

[Content	2](#_Toc188793068)

[1.	Supporting text S1. Detailed methods and materials	3](#_Toc188793069)

[1.1.	Bioinformatic analysis	3](#_Toc188793070)

[1.2.	Statistical tests	3](#_Toc188793071)

[2.	Supplemental References	4](#_Toc188793072)

# 1. Supporting text S1. Detailed methods and materials

## 1.1. Bioinformatic analysis

We analyzed the published, high-continuity genomes of 19 fungal species and strains (Tables S1, S2, S3) for differences in gene-wise relative synonymous codon usage (RSCU) between core and accessory chromosomes, as determined by the BioKIT bioinformatics tool (Steenwyk *et al.*, 2022). Please note that fungal genomes with highly fragmented assemblies were not included in the analysis, as the categorization of accessory or core chromosomes could be erroneous. In addition, genes on contigs that, based on the available information, could not be classified as either accessory or core chromosomal were excluded from the analysis of the gene-wise RSCU.

Transposable elements (TEs) were identified and soft-masked using EDTA (v2.2.0) (Ou *et al.*, 2019) with the following command:

EDTA.pl -t 32 -genome "$genome_fasta_file" -species others --anno 1 --sensitive 1 --force 1

TEs were soft-masked using Bedtools (v2.26.0), and this soft-masked genome was used with Braker3 (v3.0.8) ) (Gabriel *et al.*, 2024) for *de novo* gene annotation, incorporating protein evidence from OrthoDB11/Fungi.fa with the following command:

braker.pl --fungus --genome="$softmasked_genome_fasta_file" --species="$species" --prot_seq=OrthoDB11/Fungi.fa --workingdir="${wd}" --gff3

The resultant GFF3 file was used with gffread (v0.12.07) to extract the coding sequences using the following command:

gffread -g "$softmasked_genome_fasta_file" -x "$cds.fasta" "$GFF_FILE"

The resulting cds.fasta file was then used with BioKIT (v0.1.3) (Steenwyk *et al.*, 2022) to determine gene-wise relative synonymous codon usage, with the following command:

biokit gene_wise_relative_synonymous_codon_usage "$cds.fasta" > "${RSCU_DIR}/${species}.cds.genewise.codonusage"

## 1.2. **Statistical** tests

All graphical depictions of the data and statistical analyses were conducted in R (4.3.1) (Team, 2013) using RStudio (2023.06.0). For each strain and species, gwRSCU values were compared between those located on core chromosomes and those located on accessory chromosomes using the Wilcoxon rank-sum test. The p-values were adjusted for multiple testing using the Hochberg correction. Cliff’s delta (d) was calculated using the effsize library.

# 3. Supplemental References

Gabriel L, Brůna T, Hoff KJ, Ebel M, Lomsadze A, Borodovsky M, *et al.* (2024). BRAKER3: Fully automated genome annotation using RNA-seq and protein evidence with GeneMark-ETP, AUGUSTUS, and TSEBRA. *Genome Res* **34**: 769–777.

Ou S, Su W, Liao Y, Chougule K, Agda JRA, Hellinga AJ, *et al.* (2019). Benchmarking transposable element annotation methods for creation of a streamlined, comprehensive pipeline. *Genome Biology* **20**: 275.

Steenwyk JL, Buida TJ III, Gonçalves C, Goltz DC, Morales G, Mead ME, *et al.* (2022). BioKIT: a versatile toolkit for processing and analyzing diverse types of sequence data. *Genetics* **221**: iyac079.

Team RC (2013). R: A language and environment for statistical computing.
